# Electrophysiological Correlates of Subliminal Perception of Facial Expressions in Individuals with Autistic Traits: A Backward Masking Study

**DOI:** 10.3389/fnhum.2017.00256

**Published:** 2017-05-23

**Authors:** Svjetlana Vukusic, Joseph Ciorciari, David P. Crewther

**Affiliations:** Centre for Human Psychopharmacology, Swinburne University of TechnologyMelbourne, VIC, Australia

**Keywords:** autism, backward masking, subliminal facial emotions, subconscious, EEG

## Abstract

People with Autism spectrum disorder (ASD) show difficulty in social communication, especially in the rapid assessment of emotion in faces. This study examined the processing of emotional faces in typically developing adults with high and low levels of autistic traits (measured using the Autism Spectrum Quotient—AQ). Event-related potentials (ERPs) were recorded during viewing of backward-masked neutral, fearful and happy faces presented under two conditions: subliminal (16 ms, below the level of visual conscious awareness) and supraliminal (166 ms, above the time required for visual conscious awareness). Individuals with low and high AQ differed in the processing of subliminal faces, with the low AQ group showing an enhanced N2 amplitude for subliminal happy faces. Some group differences were found in the condition effects, with the Low AQ showing shorter frontal P3b and N4 latencies for subliminal vs. supraliminal condition. Although results did not show any group differences on the face-specific N170 component, there were shorter N170 latencies for supraliminal vs. subliminal conditions across groups. The results observed on the N2, showing group differences in subliminal emotion processing, suggest that decreased sensitivity to the reward value of social stimuli is a common feature both of people with ASD as well as people with high autistic traits from the normal population.

## Introduction

Autism spectrum disorder (ASD) is a neurodevelopmental disorder characterized by difficulties in reciprocal social interaction and a restricted range of interests and behaviors (American Psychiatric Association, 2000, 2013). It has been proposed that individuals with ASD have difficulties in the processing of facial expressions, particularly for complex or negative emotional expressions (Adolphs et al., 2001; Castelli, 2005; Golan et al., 2007). However, this finding is not uniform (e.g., Ozonoff et al., 1990; Piggot et al., 2004; Castelli, 2005). Several theoretical models have been proposed for explaining atypical socio-emotional behavior in autism (see reviews, Gaigg, 2012; Hamilton, 2013), and one of them, the Amygdala Theory of Autism, suggests that dysfunction or damage of the amygdala region leads to social impairments in autism (Baron-Cohen et al., 2000). Although there is substantial experimental support for atypical amygdala activation in individuals with autism (e.g., Critchley et al., 2000; Ashwin et al., 2006, 2007), some studies have questioned whether the amygdala plays a specific role of emotional recognition in autism (e.g., Paul et al., 2010; Birmingham et al., 2011; also see Zalla and Sperduti, 2013). Recently, impairments in functional networks as an explanation for socio-cognitive difficulties in autism have been receiving more support in the literature than dysfunction of a single brain region (Kana et al., 2011). For example, Di Martino et al. (2009) showed that autistic traits in neurotypical adults were related to the pregenual anterior cingulate cortex connectivity with insula, and more specifically, this connectivity being limited to the anterior mid-insula rather than anterior insula *per se*. Previous studies showed the importance of those regions for social function, particularly emotional processing, with the pregenual anterior cingulate cortex playing a crucial role in thinking about others’ thoughts and beliefs (Amodio and Frith, 2006; Gilbert et al., 2006), while insula is involved in the processing of sensations and emotions (Singer, 2006).

The amygdala has a central role in the processing of facial emotions in healthy populations. Although it is considered to respond primarily to threatening facial expressions, especially of fearful faces (Morris et al., 1996), it was also found to be involved in the processing of non-threatening facial expressions, such as happy and sad faces (Breiter et al., 1996; Wright et al., 2002; Yang et al., 2002). The amygdala is considered to have an essential role in a vigilance system for rapidly alerting other brain regions to the importance of social stimuli (LeDoux, 1996; Whalen, 1998; Schultz et al., 2000), suggesting that in autism, if amygdala function is disrupted, faces may be less socially salient, leading to reduced experience with emotional facial stimuli (Schultz et al., 2000; Hall et al., 2007). Based on those findings, several additional theoretical models of autism have been proposed in order to explain socio-emotional difficulties in autism. For example, the Relevance Detection Theory (Sander et al., 2003) suggests that the amygdala is part of an extended cortico-limbic system that is important for recognizing cues in environmental stimuli that immediately orient a person towards dangers or safety in the environment. In addition, the Social Motivation Theory of autism (Dawson et al., 2005) has proposed that abnormal social motivation prevents orienting to socially relevant stimuli including faces. This suggests that a lack of experience with faces during critical developmental periods in individuals with autism, leads to difficulties in expert and automatic processing of faces.

The amygdala can be engaged subconsciously by presenting images of facial emotions with very short duration and masking them (Morris et al., 1998; Whalen et al., 1998; Liddell et al., 2005), rendering them outside of conscious awareness or by presenting them under conditions of binocular suppression (Williams et al., 2004). One popular, although not universally accepted (see Pessoa and Adolphs, 2010), model proposes that fear-related responses are processed through a direct subcortical pathway comprising the amygdala, the superior colliculus and the pulvinar nucleus of the thalamus, for fast, but coarse analysis of potential threat that has adaptive survival value (LeDoux, 1996, 2000; Vuilleumier et al., 2003; Tamietto and de Gelder, 2010; Tamietto et al., 2012).

Research support for subliminal processing of emotional faces has been found in studies of affective subliminal priming (Murphy and Zajonc, 1993; Monahan et al., 2000; Nomura et al., 2004; Finkbeiner and Palermo, 2009; Jiang et al., 2013), and with cortically blind patients (de Gelder et al., 1999). For example, de Gelder et al. (1999) showed that a patient with right hemianopia, or blindness in the right visual field due to damage to his left occipital lobe, was able to guess the facial emotional expression at a level above chance, even if stimuli were not seen consciously.

An important research method that is often used to examine subliminal automatic responses is the backward masking paradigm (for a review of this paradigm, see Breitmeyer and Ogmen, 2000). Esteves and Öhman (1993) and Öhman and Soares (1994) were among the first researchers to use this paradigm in emotion research, with Öhman and Soares (1994) using common phobic objects, such as snakes, as stimuli, whereas Esteves and Öhman (1993) used emotional faces as stimuli. In the backward masking paradigm, there is a very brief presentation of the face stimuli followed by a mask that blocks the conscious recognition of a stimulus. The interval between the onset of the target and masking stimuli, the stimulus-onset-asynchrony (SOA), was the principal factor in correctly perceiving the target stimulus within this paradigm (Esteves and Öhman, 1993).

EEG backward-masking studies have demonstrated an ability to measure temporal processing of emotional stimuli presented below the level of visual awareness in healthy participants. Liddell et al. (2004) suggested that subliminal and supraliminal emotion processing could be distinguished with the N2/early P3 components representing orienting and N4/late P3 event integration, based on the Halgren and Marinkovic (1995) model of emotion processing. The results of their study supported this model, showing larger the N2 amplitude and faster P3a latencies for subliminally presented fearful faces compared to neutral faces, and larger P3b amplitude in response to supraliminal fearful faces (Liddell et al., 2004). This indicates that event-related potential (ERP) components can be used to show a double dissociation for subliminal vs. supraliminal processing of fearful facial expressions (Liddell et al., 2004). The finding of enhanced N2 for subliminal fearful faces was initially criticized by Pegna et al. (2008) for using a passive viewing task, citing Kiss and Eimer (2008) who suggested that the passive task may prevent participants from attending to masked stimuli. Contrary to Liddell et al. (2004), Pegna et al. (2008) found an increased N2 for fearful compared to non-fearful faces at longer durations of presentation (supraliminal rather than subliminal condition) by using an active emotion recognition task. However, a subsequent study (Pegna et al., 2011) indicated that a difference between subliminally presented fearful and non-fearful faces can be observed on both the early N170 component and also elicit posterior positivity and fronto-central negativity at around 200–250 ms, even when participants’ attention is engaged in an incidental task. Thus, the N2 peak is a component worth observing with regard to subliminal emotion processing. Following these results, the present study investigated both the N2/P3a and the N4/P3b while processing emotion.

However, several EEG backward-masking studies have reported earlier emotional differentiation in the subliminal condition. For example, Kiss and Eimer (2008) and Eimer et al. (2008) found an enhanced frontal positivity for both subliminal and supraliminal fearful faces compared to neutral faces between 140 ms and 180 ms post-stimulus. Pegna et al. (2008) examined processing of fearful and non-fearful (happy and neutral) faces at subliminal, intermediate and supraliminal stimulus durations of 16 ms, 33 ms, 66 ms, 133 ms and 266 ms. This study found emotion discrimination for subliminal faces in the N170 component, showing increased N170 responses to fearful compared to non-fearful faces for all three durations (subliminal, intermediate and supraliminal) over temporal electrodes.

The N170 is considered a face-specific component reflecting structural encoding of faces and represents the earliest stages of face processing (Bentin et al., 1996; Itier and Taylor, 2004). As there is evidence of atypical responses on the N170 ERP component in children and adults with autism compared to those without autism (e.g., McPartland et al., 2004; O’Connor et al., 2005, 2007; Hileman et al., 2011), component was also included in the design of the present study. There is mixed evidence about modulation of the N170 by facial expressions in both typically developing subjects (e.g., Batty and Taylor, 2003, 2006; Blau et al., 2007; Eimer et al., 2008; Luo et al., 2010) and subjects with autism (Dawson et al., 2004; O’Connor et al., 2005; Wong et al., 2008; Batty et al., 2011).

While there are several behavioral reports of impaired processing of briefly presented emotional faces in autism (e.g., Kamio et al., 2006; Hall et al., 2007), there is a relative lack of EEG studies on automatic face processing in the disorder. Recently, Fujita et al. (2013) measured visual evoked potentials (VEPs) elicited by subliminally presented fearful and neutral faces and objects in the upright and inverted position and found group differences between individuals with ASD and typically developing individual in the earliest VEP component (N1), indicating altered early visual processing of briefly presented emotional faces in the disorder.

Backward masking has been used in several fMRI studies investigating subliminal processing of emotional and social information in autism. While deficits were found in subliminal processing in ASD (Kamio et al., 2006; Hall et al., 2007; Kleinhans et al., 2011), other studies did not find differences between subjects with autism and healthy controls in the amygdala activation during sub-threshold presentation of facial expressions (Hall et al., 2010). However, additional evidence for atypical subliminal face processing in autism is found in psychophysiological studies using facial electromyography (EMG). Facial EMG studies with neurotypical subjects suggest that the observation of others’ emotional facial expressions automatically produces similar facial expressions or facial mimicry in the observer (Sato et al., 2013). However, atypical automatic facial mimicry to backwardly masked briefly presented happy and angry facial expressions was found in adults with ASD (Mathersul et al., 2013). Impaired recognition of briefly presented (but not backwardly masked) happy and angry faces was also found in young adults with ASD (Clark et al., 2008). Studying facial emotion processing in autism is important because they are crucial for social functioning and social communication (Grelotti et al., 2002; Dawson et al., 2005; Golan et al., 2007). In addition, understanding of automatic emotion processing in the disorder can provide better understanding and clarification of specificity of emotion processing in this group. The importance of understanding various aspects of emotion processing is particularly evident from a recent study (Tseng et al., 2016) that showed differences in neural activity for arousal but not valence on emotion processing between subjects with autism and neurotypically developing subjects.

The aim of the present study was to assess, using EEG, the processing of subliminal and supraliminal fearful and happy facial expressions by investigating differences in high and low autistic traits as measured by the Autism Spectrum Quotient (AQ; Baron-Cohen et al., 2001). Following the model (Liddell et al., 2004) that considers N2/early P3 components representing “orienting” and N4/late P3 “event integration”, our task was to investigate both sets of ERPs. Based on those findings, we predicted more prominent activity over N2 and P3a (early P3) components as a response to subliminal fearful and happy facial stimuli, and more prominent activity over N4 and P3b (late P3) ERP components as a response to supraliminal fearful and happy facial stimuli. We suggest that, based on previous fMRI findings of deficits in subliminal processing in autism (Kamio et al., 2006; Hall et al., 2007; Kleinhans et al., 2011), we would find group differences predominantly in subliminal condition (or orienting stages of face processing). This stage of emotion processing is important for creation of conscious emotional experience.

The investigation of individuals with high and low autistic traits can have some advantages compared to working with individuals with autism, particularly related to heterogeneity of autism and differing diagnostic criteria used. In research with facial emotional stimuli, this group of participants can exclude differences due to extensive training with face stimuli that individuals with autism can have through various intervention programs (e.g., Herbrecht et al., 2009), and that are rarely mentioned in research studies on face processing.

## Materials and Methods

### Participants

The participants were selected from a total population of 94 individuals who completed online surveys including the AQ and the Empathy Quotient (EQ). Based on their AQ scores, 26 participants (all right-handed, 13 females) were selected to participate in the EEG study (see Table 1). The selection procedure consisted of creating two groups of participants: those who achieved scores ≤10 (Low AQ) and ≥22 (High AQ) on the AQ questionnaire (total sample *N* = 94, median score = 15, SD = 7.8). The AQ cut off scores in this study are similar to some other studies (e.g., Sutherland and Crewther, 2010). Demographic information of participants was also collected online. All participants had a normal or corrected-to-normal vision, with no neurological impairment (including clinical autism). They gave informed signed consent to participate, and all experimental procedures were approved by the Swinburne University Human Research Ethics Committee (SUHREC). All participants volunteered to be part of the study. Participants consented to the study after carefully reading the Consent forms for this study, which were approved by the SUHREC. No vulnerable populations were involved.

**Table 1:** **Participant group characteristics**.

AQ group		AQ score	EQ score	Raven’s	Age
Low (*n* = 14; 6 females)	Mean	7.86	53	21.64	28.57
	SD	2.8	9.72	5.03	6.15
	Minimum	2	41	12	19
	Maximum	11	70	31	43
High (*n* = 12; 7 females)	Mean	25.25	35.67	19.92	30.58
	SD	5.51	13.01	6.82	10.3
	Minimum	21	10	10	19
	Maximum	39	59	31	55

### Measures

All participants completed online questionnaires: the AQ and the EQ. Participants also completed the Advanced Raven’s Progressive Matrices before or after EEG testing.

The AQ is a self-administered questionnaire that consists of 50 questions, devised to quantitatively measure the degree to which a person with normal intelligence has autistic traits (Baron-Cohen et al., 2001; Woodbury-Smith et al., 2005). Participants respond using a 4-point rating scale, from “definitely agree” to “definitely disagree”.

The EQ is a self-administered questionnaire that consists of 40 questions assessing empathy (Baron-Cohen and Wheelwright, 2004) with high test-retest reliability (Lawrence et al., 2004). Lower scores on the EQ have been found in adults with autism (Baron-Cohen and Wheelwright, 2004) and in neurotypical men compared to women (Baron-Cohen and Wheelwright, 2004; Lawrence et al., 2004).

The Raven’s Advanced Progressive Matrices (RAPM) is a standardized nonverbal intelligence test, and is generally used as a test of general cognitive ability and intelligence (Raven, 2000). It consists of visually presented geometric figures where one part is missing and the missing part must be selected from a panel of suggested answers to complete the designs. In the present study, we used the RAPM with a time limit of 20 min, to eliminate general intelligence as a potential explanation of any differences found between AQ groups.

### Stimuli

The stimuli consisted of grayscale photographs of the faces of 18 Caucasian models (nine male, nine female). The models’ faces depicted neutral, fearful and happy expressions (with both open and closed mouth exemplars) and were cropped with an oval shape, removing external features. The facial images were taken from the NimStim set (Tottenham et al., 2009), and masks were created by phase scrambling images of neutral faces using MatLab. The use of phase-scrambled images as a mask was based on past neuroimaging studies (e.g., Jacques and Rossion, 2007; Rousselet et al., 2007; Schultz and Pilz, 2009) that have used phase-scrambled stimuli because they make faces unrecognizable without altering their original power spectrum, luminance and contrast.

### Experimental Procedure

**Figure 1:**
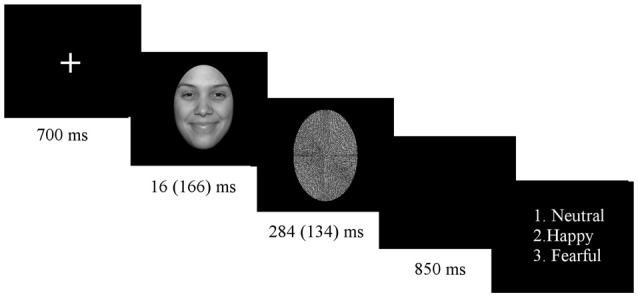
**Experimental procedure**.

Subjects sat in an electrically shielded, dimly-lit and sound-attenuated room in front of a computer screen. The experiment was programmed with E-Prime 1.2 (Psychology Software Tools, Inc., Pittsburgh, PA, USA). Stimuli were presented in 8 blocks of 138 trials, each block consisting of a randomized presentation of both subliminal and supraliminal faces. Block order was counterbalanced across participants. Before starting the experimental procedure, participants were given a practice run. At the beginning of the experiment, a white fixation cross appeared in the middle of the screen, lasting for 700 ms. Shortly thereafter, a picture of a face stimulus was displayed for duration of 16 ms (subliminal condition) or 166 ms (supraliminal condition), immediately followed by the mask for 284 ms for subliminal presentation or 134 ms for supraliminal presentation, in order to keep the presentation time constant for 300 ms (see Figure 1). Stimuli were presented on a 24″ color LCD monitor driven at 100 Hz vertical refresh rate.

At the end of each trial, a question appeared on the screen asking for explicit emotion recognition, showing numbers 1–3 that were put next to the written label for each of the three facial expressions, with a new trial started only after participant response. Participants were allowed unlimited time to press an answer and were asked to always press the answer with the right hand. In the case of subliminal stimuli (stimuli showed below the threshold of visual awareness), participants were asked to guess the facial expression. The explicit recognition task was adopted because it gives equal importance to all facial expressions in both conditions. There was an equal number of trials in each condition for each facial expression (120 trials for each facial expression, for each condition).

### Electrophysiological Recording

EEG activity was recorded using a Neuroscan amplifier (Compumedics USA, Charlotte, NC, USA) from 64 electrodes, placed in accordance with the International 10–20 system. The amplification was set at 1000× EEG signals were band pass filtered 0.05–70 Hz with a sampling rate of 500 Hz. Electrode impedances were kept below 5 kΩ. The vertex (Cz) electrode was used as a reference. Recordings were re-referenced to the average reference as computed from all scalp electrodes (for the N170 component), and to the average of mastoids (for N2, P3a, P3b, N4). This re-referencing method of the average of mastoids used for the latter components was chosen because it is used most frequently for endogenous ERP components. However, mastoid re-reference can be problematic for the N170 because this ERP is usually largest over lateral posterior regions, close to mastoids (Luck, 2005). It was found that the common average reference yielded the largest N170 amplitude and the smallest amplitude at the vertex positive potential (VPP; Joyce and Rossion, 2005). On the other hand, it has been suggested that using a mastoid reference eliminates the specificity of the N170 response, yielding the smallest peaks at the N170 sites and the largest peaks at VPP sites (Schendan et al., 1998; Rossion et al., 2003; Joyce and Rossion, 2005). EOG was recorded from two electrodes placed at the external canthi of both eyes and from two electrodes on the infraorbital and supraorbital areas of the left eye to monitor for eye movements and blinks. The raw data were segmented into epochs of −200 to 800 ms around stimulus events. Trials in which the amplitude exceeded ±100 μV were automatically rejected, eliminating eye blinks and other movements. Trials with EOG artifacts (e.g., eye blinks or large eye movement) were also removed, with additional artifacts excluded upon visual inspection. Only participants with more than 50% of artifact-free trials in each condition were included in the final analysis. ERPs were averaged separately for each stimulus category (each emotion was averaged for subliminal and supraliminal threshold conditions), baseline corrected and low-pass filtered at 30 Hz (24 dB/octave).

### Behavioral Analysis

Independent samples *t*-tests were conducted to compare groups on EQ scores and RAPM.

In the behavioral analysis we reported the accuracy rates and due to unlimited time given to participants for recognizing facial expressions, we did not report the reaction times (RTs).

The behavioral analysis consisted of a series of one tailed *t*-tests comparing accuracy to chance levels for the two conditions and for each of the emotions within the conditions. This was followed by the repeated measures ANOVA with emotion and condition as a within-subjects factors and the AQ group as a between-subjects factor.

Additional estimates of detection measure (*d′*) and response bias (*c*) (Macmillan and Creelman, 1991) were computed separately for each participant for each of the facial expressions in each condition. The signal detection is considered a particularly suitable behavioral analysis for the experimental procedure where participants need to guess as it shows inflated hit rates. Contrary to this method, the response accuracy percentage is known to be highly sensitive to response bias (Macmillan and Creelman, 1991). We performed repeated measures ANOVAs for the *d′* values and the response bias *c* with emotion and condition as a within-subjects factors and the AQ group as a between-subjects factor.

### ERP Analyses

The time windows for ERP components and grand averages were selected based on previous literature (Liddell et al., 2004; Kiss and Eimer, 2008; Pegna et al., 2008). The N170 ERP was examined at the lateral occipito-temporal sites P7 and P8, and N2, P3a, P3b and N4 components were examined at midline electrodes Fz, Cz, and Pz. The peak amplitudes and latencies were measured in the following latency windows: N170 (140–220 ms), N2 (180–300 ms), P3a (240–350 ms), P3b (400–700 ms) and N4 (300–500 ms). The amplitude and latency of each ERP component were quantified by the highest peak value within the chosen latency window.

ERP amplitude and latency were analyzed with repeated-measures ANOVA using the AQ Group as the between-subject factor, with emotion (neutral, happy, fearful), condition (subliminal, supraliminal), and hemisphere (left and right, only for N170)/electrode (Fz, Cz, Pz) as within-subject factors. Degrees of freedom were adjusted (Greenhouse-Geisser epsilon) for factors with greater than two levels. Paired-samples *t*-tests were performed to supplement the ERP findings. An alpha criterion level of 0.05 was used unless otherwise noted.

## Results

### Behavioral Results

The independent samples test was used to compare groups on EQ scores. The study found significantly higher EQ scores for the Low AQ group (EQ = 53.50) compared to the High AQ group (EQ = 35.67; see Table 1), (*t*_(24)_ = 3.995, *p* = 0.001). This result, indicating lower scores on the self-report empathy test in typically developing individuals with higher AQ, supplements previous evidence showing that the empathy difficulties, indicated by lower EQ scores, are also found in parents of children with autism, particularly fathers (Sucksmith et al., 2013). Sucksmith et al. (2013) suggested that lower scores on the EQ test may represent a reliable feature of the broad autism phenotype in fathers.

**Figure 2:**
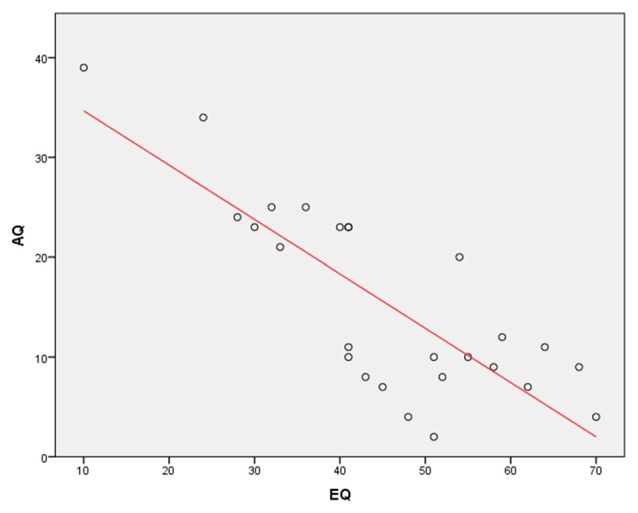
**A Pearson’s correlation between Autism Spectrum Quotient (AQ) and Empathy Quotient (EQ) scores**. The results show a very strong, negative correlation between the AQ and EQ (*r* = −0.81, *p* < 0.0001).

In addition, a Pearson’s correlation was run to determine the relationship between AQ and EQ scores. The results showed a very strong, negative correlation between the AQ and EQ (*r* = −0.81, *N* = 26, *p* < 0.0001; Figure 2). This finding again complements a previous research showing that the EQ is inversely correlated with the AQ (Baron-Cohen and Wheelwright, 2004).

There was no evidence for between-group differences on the RAPM (*t*_(24)_ = 0.742, *p* = 0.47).

The analysis revealed an accuracy rate of 57.1% in the subliminal and 92.6% in the supraliminal conditions (above chance (33.3%) accuracy rate for both conditions, both *p* = 0.0001). Response accuracy was above chance (33.3%) for all facial expressions in supraliminal condition (all* p* < 0.000). For facial expressions in subliminal condition, accuracy rates were above chance for neutral and fearful faces (*t*_(25)_ = 8.74, *p* = 0.0001 [neutral] and *t*_(25)_ = 6.04, *p* = 0.0001 [fearful]), but it was below chance for happy faces (*t*_(25)_ = 1.75, *p* = 0.09). Table 2 shows mean accuracy results as percentages across both groups and for each AQ group.

**Table 2:** **Accuracy rates (%) across both groups (*N* = 26) and for Low AQ (*N* = 14) and High AQ (*N* = 12) groups**.

	**All groups**		**Low AQ**		**High AQ**	
	Mean	SD/SE	Mean	SD/SE	Mean	SD/SE
Accuracy (subliminal) (%)	57.08	13.04/2.56	57.26	15.33/4.1	56.86	10.42/3.01
Accuracy (supraliminal) (%)	92. 64	4.15/0.81	93.81	3.32/0.89	91.28	4.73/1.37
Accuracy (neutral subliminal) (%)	67.18	19.76/3.88	70.18	19.98/5.34	63.68	19.77/5.71
Accuracy (happy subliminal) (%)	40.93	22.24/4.36	42.14	26.28/7.02	39.51	17.43/5.03
Accuracy (fearful subliminal) (%)	62.82	24.91/4.88	59.29	28.99/7.75	66.94	19.55/5.64
Accuracy (neutral supraliminal) (%)	94.04	5.48/1.07	95.06	4.06/1.09	92.85	6.76/1.95
Accuracy (happy supraliminal) (%)	91.60	4.12/0.81	92.2	3.53/0.94	90.9	4.79/1.38
Accuracy (fearful supraliminal) (%)	92.24	7.94/1.56	94.05	6.14/1.64	90.14	9.47/2.73

*Results show both subliminal and supraliminal conditions*.

An additional analysis applied the repeated measures ANOVA with emotion and condition as a within-subjects factors and the AQ group as a between-subjects factor. The results showed significant effect of Condition (*F*_(1,24)_ = 255.5, *p* = 0.0001), indicating higher accuracy rates in the supraliminal condition. The results also found significant effects of Emotion (*F*_(2,38)_ = 9.45, *p* = 0.001 and Emotion × Condition interaction (*F*_(2,39)_ = 10.16, *p* = 0.001). The paired *t*-tests showed lower accuracy rates for happy compared to neutral and fearful faces across both conditions (all *p* = 0.001; 66.19% for happy, 77.6% for fearful and 88.44% for neutral faces. We also applied the repeated measures ANOVA on each condition and found a significant effect of Emotion (*F*_(2,38)_ = 10.23, *p* = 0.001) in the subliminal condition, with happy faces showing lower accuracy rates compared to neutral (*p* = 0.002) and fearful (*p* = 0.0001) facial expressions (40.93% for happy, 62.82% for fearful and 67.18% for neutral faces). There was no main effect of AQ group for accuracy rates.

Across both AQ groups, the *d′* values for trials in both conditions were significantly greater than zero (in subliminal condition *d′* was 1.23 (SD = 0.66), *t*_(25)_ = 9.55, *p* = 0.0001; in supraliminal condition the *d′* values were 4.12, (SD = 0.9), *t*^(25)^ = 23.39, *p* = 0.0001), suggesting a non-random response on both conditions.

The repeated measures ANOVA was applied for the *d′* values. The analysis revealed the main effect of Condition (*F*_(1,24)_ = 445.82, *p* = 0.0001), indicating larger *d′* values for supraliminal than subliminal condition. In addition, significant effects of Emotion (*F*_(2,37)_ = 25.18, *p* = 0.0001) and Emotion × Condition interaction (*F*_(2,48)_ = 7.88, *p* = 0.001) were also found. The paired *t*-test indicated significant differences between all emotions, particularly showing larger *d′* values for happy compared to neutral (*p* = 0.0001) and fearful faces (*p* = 0.01) and larger *d′* values for fearful compared to neutral (*p* = 0.0001) faces. When each condition was analyzed separately, the results showed a main effect of Emotion (*F*_(2,48)_ = 9.31, *p* = 0.0001) in the subliminal condition, showing lower *d′* values for neutral faces compared to happy (*p* = 0.01) and fearful faces (*p* = 0.0001).

The repeated measures ANOVA was also applied for the response bias *c*. The results showed significant effects of Condition (*F*_(1,24)_ = 62.72, *p* = 0.0001), with larger *c* for subliminal (mean = 0.391) than supraliminal condition (mean = 0.172). The main effects of Emotion (*F*_(2,37)_ = 7.7, *p* = 0.003) and Emotion × Condition interaction (*F*_(2,39)_ = 10.62, *p* = 0.001) were also found. The paired *t*-test revealed the higher *c* for happy compared to neutral (*p* = 0.001) and fearful faces (*p* = 0.03). An additional analysis for each condition revealed a main effect of Emotion (*F*_(1,33)_ = 13.27, *p* = 0.0001) in the subliminal condition, with higher *c* for happy compared to neutral and fearful faces (all *p* = 0.0001). There were no significant Emotion effects in the supraliminal condition. These results indicate that participants were less likely to report happy faces than other faces in subliminal condition.

There was no main effect of AQ group either for the detection *d′* values or response bias *c*.

### ERP Results

The planned analysis for N2, P3a, P3b and N4 ERP components included analysis both across all regions and for each midline region. The final ERP analysis included both correct and incorrect behavioral responses.

#### N2 Amplitude and Latency

The analysis of the N2 amplitude data showed a significant effect of Region (*F*_(2,38)_ = 30.35, *p* = 0.0001), indicating that the N2 was larger in the frontal (Fz) than central (Cz) and parietal (Pz) regions (both *p* = 0.0001) and also larger in the central (Cz) than parietal (Pz) (*p* = 0.02) region.

**Figure 3:**
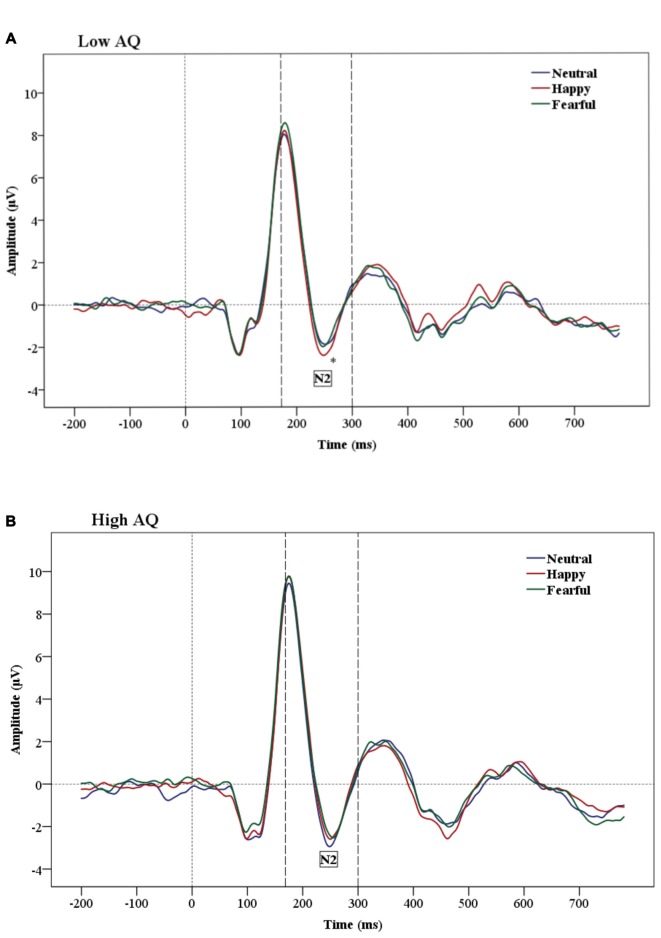
**Grand-average Event-related potential (ERP) waveforms of N2 amplitudes for Low AQ (A)** and High AQ **(B)** groups (across all regions). The Low AQ group shows larger amplitudes for happy than neutral faces in the subliminal condition. The High AQ group does not show emotional differentiation.

**Figure 4:**
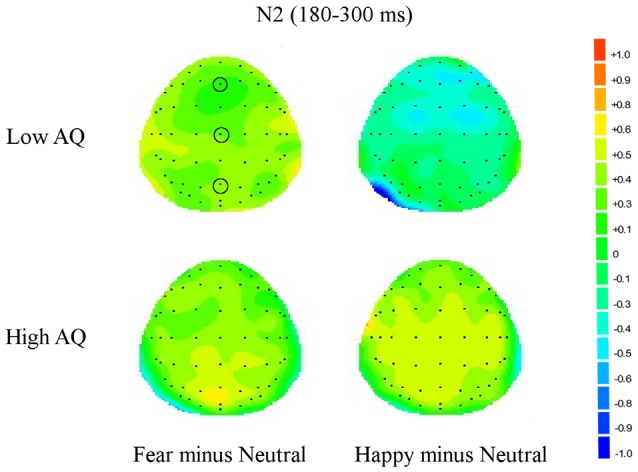
**Topographic maps for subliminal faces**. Topographic maps show the cortical activities during 180–300 ms (N2) for subliminally presented fearful minus neutral and happy minus neutral faces in Low and High AQ groups. An increased negativity (blue color) is found for the Low AQ group for happy minus neutral faces predominantly in fronto-contral regions.

The results of N2 amplitude analysis showed a significant Emotion × Condition × AQ (*F*_(2,47)_ = 3.54, *p* = 0.04) interaction. Separate ANOVAs were performed for each condition and a significant Emotion by AQ group interaction (*F*_(2,48)_ = 5.2, *p* = 0.01) was found only in the subliminal condition. Further analysis showed a main effect of Emotion in the Low AQ (*F*_(2,26)_ = 3.52, *p* = 0.05), with larger N2 amplitudes for happy compared to neutral faces (*p* = 0.03). No significant effects were found for N2 amplitudes for the High AQ group (see Figures 3A,B, 4).

The N2 latency analysis showed a main effect of Condition (*F*_(1,24)_ = 28.01, *p* = 0.0001), indicating that subliminal stimuli elicited shorter N2 latencies than supraliminal stimuli.

#### P3a Amplitude and Latency

No significant effects were found for P3a amplitudes.

The P3a latency analysis showed a main effect of Condition (*F*_(1,24)_ = 18.15, *p* = 0.0001), indicating significantly shorter P3a latencies in the supraliminal than in the subliminal condition.

The results for P3a latencies also showed a significant Emotion × Condition × Region interaction (*F*_(4,96)_ = 2.46, *p* = 0.05). This interaction was further examined by conducting an additional ANOVA for each region and results showed an Emotion × Condition interaction (*F*_(1,48)_ = 3.62, *p* = 0.05) in the frontal (Fz) region. Further analysis in this region found a main effect of Emotion (*F*_(2,38)_ = 3.76, *p* = 0.04) in the supraliminal condition, with a shorter P3a latency for happy compared to neutral faces (*p* = 0.05). In addition, there was a significant effect of Emotion (*F*_(2,48)_ = 3.16, *p* = 0.05) in the central (Cz) region indicating shorter P3a latencies for happy compared to neutral faces (*p* = 0.05) across both conditions.

#### P3b Amplitude and Latency

The main effect of Condition was significant for both P3b amplitudes (*F*_(1,24)_ = 17.2, *p* = 0.0001) and P3b latencies (*F*_(1,24)_ = 28.63, *p* = 0.0001), indicating larger amplitudes and shorter latencies in the supraliminal than in the subliminal condition.

The results of P3b latencies showed a significant Condition × Region × AQ interaction (*F*_(2,48)_ = 7.67, *p* = 0.001). Further analysis found the main effect of Condition (*F*_(1,13)_ = 10.1, *p* = 0.007) in the frontal (Fz) region and in the Low AQ group only, indicating a significantly shorter P3b latency in the supraliminal than in the subliminal condition. There was no significant effect of Condition in the frontal (Fz) region in the High AQ group (*F*_(11)_ = 1.62, *p* = 0.23).

#### N4 Amplitude and Latency

There were no significant main effects for N4 amplitudes.

The analysis for the N4 latency found a significant Condition × AQ interaction (*F*_(1,24)_ = 4.83, *p* = 0.04). Separate ANOVAs for each group found a significantly shorter N4 latency under supraliminal than under subliminal condition only in the Low AQ group (Condition effect; *F*_(1,13)_ = 7.53, *p* = 0.02). The Low AQ group also showed a significant Condition × Region interaction (*F*_(2,26)_ = 5.42, *p* = 0.02), and further analysis indicated that the shorter N4 latency under the supraliminal condition is mostly observed in frontal (Fz; *F*_(1,13)_ = 12.43, *p* = 0.004), and central (Cz; *F*_(1,13)_ = 5.6, *p* = 0.03) regions.

Statistical significant differences between subliminal and supraliminal condition across all participants and for each ERP can be seen in the Table 3.

**Table 3:** **Peak latencies (ms) of event-related potential (ERP) components**.

	All subjects (*n* = 26)		
	Subliminal	Supraliminal	
	Latency (ms)/SD	Latency (ms)/SD	*P* value
**N170**			
(P7, P8)	184.9 (18.3)	180.3 (12.4)	0.01**
P7	183.8 (20.4)	180.3 (15.4)	0.05*
P8	186.0 (16.2)	180.2 (8.4)	0.01**
**N2**			
(Fz, Cz, Pz)	253.3 (24.9)	272.5 (25.0)	0.0001***
Fz	260.4 (15.3)	279.6 (19.2)	0.0001***
Cz	259.1 (19.2)	274.4 (18.3)	0.001***
Pz	240.4 (40.1)	263.4(37.6)	0.003**
**P3a**			
(Fz, Cz, Pz)	314.0 (37.5)	284.5 (43.2)	0.0001***
Fz	322.2 (37.5)	283.3 (45.9)	0.0001***
Cz	317.4 (35.4)	286.10(44.2)	0.002**
Pz	302.4 (39.6)	284.0 (39.4)	0.04*
**N4**			
(Fz, Cz, Pz)	415.9 (72.4)	396.48 (86.7)	0.127
Fz	402.8 (79.7)	366.85 (85.7)	0.05*
Cz	415.3 (71.2)	396.44 (91.0)	0.249
Pz	429.7 (66.4)	426.15 (83.5)	0.809
**P3b**			
(Fz, Cz, Pz)	537.6 (97.0)	456.4 (81.4)	0.0001***
Fz	539.0 (103.7)	484.4 (101.4)	0.006**
Cz	548.8 (98.1)	458.0 (80.6)	0.0001***
Pz	524.9 (87.9)	426.7 (62.3)	0.0001***

Peak latencies across all participants and for all ERPs (across all electrodes and for each electrode), showing significant differences between subliminal and supraliminal conditions. Results show shorter latencies in subliminal than supraliminal condition on the N2. Shorter latencies for supraliminal than subliminal conditions are found on the N170, P3a and P3b. The N4 does not show significant differences between conditions across all three electrodes and only small difference can be seen on the frontal electrode. *p ≤ 0.05, **p ≤ 0.01, ***p ≤ 0.001.

#### N170 Amplitude and Latency

**Figure 5:**
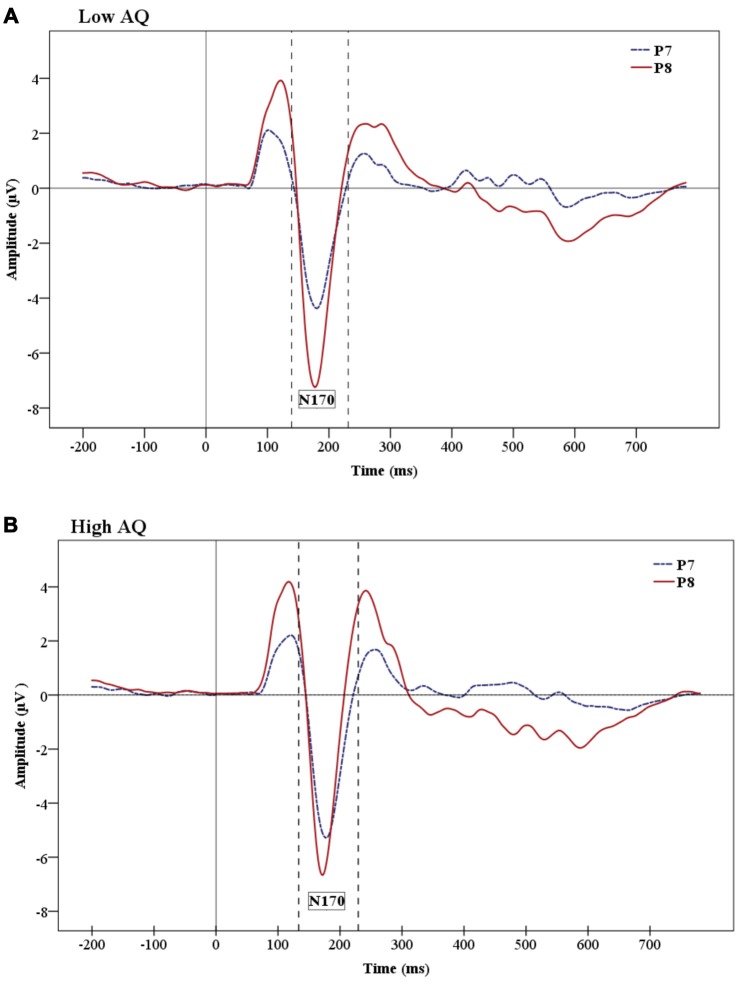
**Grand-average ERP waveforms showing the N170 ERP for Low AQ (A)** and High AQ **(B)** groups. There are larger amplitudes in the right hemisphere for both groups. However, the figures show reduced hemisphere lateralization in the High AQ.

The analysis on the N170 amplitude data showed a significant effect of Hemisphere (*F*_(1,24)_ = 9.06, *p* = 0.01), which was due to a greater negativity over the right (−7.812 μV) than the left (−5.456 μV) hemisphere (Figure 5 shows hemisphere lateralization for each group). No such effect was observed for N170 latencies.

The analysis of N170 peak latencies revealed a main effect of Condition, with shorter supraliminal than subliminal latencies (*F*_(1,24)_ = 7.36, *p* = 0.01).

No other main effects were significant for the N170.

## Discussion

The main aim of this study was to examine the processing of subliminal facial expression reflecting difference emotional states in individuals with higher and lower autistic tendencies. Partial support was found for the main hypothesis that group differences would emerge between individuals with lower and higher autistic traits in emotion effect under subliminal viewing conditions. This effect was found only for subliminally presented happy faces in the frontal region on the N2 component, showing emotional differentiation in the Low AQ group, but not in the High AQ group. No group differences were found for processing of subliminal fearful faces, contrary to our expectations. However, recently Smith (2011) examined processing of a larger number of subliminal facial expressions within a backward-masking paradigm and confirmed processing of subliminal facial expressions at the N2 without finding evidence that the emotional modulation on this component in subliminal condition is specifically related to fearful faces as found in earlier studies (Liddell et al., 2004; Kiss and Eimer, 2008). Our findings of differential emotion processing on the N2 certainly can contribute to the present discussion on subliminal processing in this component.

There are discrepancies within the backward-masking literature regarding the N2 component and emotion modulation at subliminal and supraliminal levels. Liddell et al. (2004) and Kiss and Eimer (2008) found differences between facial expressions on N2 only in the subliminal condition, whereas Pegna et al. (2008) found such differences to occur in the supraliminal condition. Some other studies found modulations in the supraliminal condition on this component by using masked line drawings as face stimuli (e.g., Wilenius-Emet et al., 2004). However, although initially considering that the N2 represents early responding to conscious emotional information, Pegna et al. (2011) in a later study, confirmed the suggestion that the N2 represent an automatic, non-conscious attention-orienting response to emotionally relevant stimuli (Liddell et al., 2004). Although the present study provides support for the emotion modulation in subliminal condition on the N2, this was found only in the Low AQ group and for happy but not for fearful faces. However, the N2 component latency was shorter for subliminal vs. supraliminal stimuli across both AQ groups.

The N2 component has been linked to the activity of the amygdala (Halgren and Marinkovic, 1995; Krolak-Salmon et al., 2004) and of anterior cingulate cortex (van Veen and Carter, 2002), adding to the importance of this component when researching autism. Both of these regions are part of the brain’s limbic system and destructive lesions of any of these regions could lead to social and communicative impairments. An updated view of the amygdala theory of autism proposes that the brain circuit, in which the amygdala occupies a crucial place, is responsible for the detection of a larger category of biologically relevant stimuli, acting as a relevance detector and giving priority to salient signals, based on the motivation and contextual goals of the perceiver (Sander et al., 2003; Zalla and Sperduti, 2013). The social relevance detector account posits that although the amygdala is able to process social information under the unaware condition, its prime role is bringing to conscious awareness salient stimuli through emotional arousal (Vuilleumier and Schwartz, 2001). Hence, according to the relevance detection theory of autism, there is reduced top-down control and attentional modulation performed by the vMPFC in this group, leading to the inability of this prefrontal area to form salience maps for giving priority to specific environmental stimuli. As happy faces have been found to activate reward circuitry in neurotypical individuals (Phillips et al., 1998; O’Doherty et al., 2003), findings in the present study may indicate decreased sensitivity to reward value of social stimuli not only in people with autism compared to typically developing controls but also in individuals with higher autistic traits compared to individuals with lower autistic traits. In this way, our results based on processing of emotional faces presented below the level of visual awareness complement studies that showed specificity in responding to happy facial stimuli in people with autism (e.g., Sepeta et al., 2012) and neurotypical participants with high AQ (e.g., Gayle et al., 2012; Sims et al., 2012).

However, it should be added that given the evidence for magnocellular dysfunction in autism (McCleery et al., 2007; Sutherland and Crewther, 2010), an inability to process visual information correctly from much earlier in cortical processing should also be taken into consideration (Kveraga et al., 2007). The magnocellular pathway is more sensitive to stimuli of lower spatial frequencies (Merigan and Maunsell, 1993), has a faster conduction speed than the parvocellular pathway (Schroeder et al., 1989; Maunsell et al., 1999) and dominates input to the dorsal cortical stream. The parvocellular pathway, on the other hand, is more sensitive to stimuli of higher spatial frequencies (Merigan and Maunsell, 1993) and conveys higher spatial frequency information to the ventral cortical visual stream (Merigan and Maunsell, 1993). Activation of the pulvinar, amygdala and superior colliculus (making a subcortical visual pathway) is restricted to low spatial frequencies but enables a fast yet coarse processing of visual information, bypassing visual cortex (Morris et al., 1996, 1999). Kveraga et al. (2007) showed that fast magnocellular pathways connect early visual and object recognition regions with the orbitofrontal cortex via the dorsal stream and, in this way, facilitate object recognition by activating early predictions about objects. They found that the amygdala receives a substantially greater magnocellular input than parvocellular input and also suggested that their finding of increased right amygdala activation for M-biased stimuli is similar to findings of greater increase of right amygdala for masked fearful faces presented below awareness level to healthy subjects (Morris et al., 1999).

Understanding the role of sensory/afferent differences is of great importance for autism research. Sutherland and Crewther (2010) nonlinear flash VEP study showed atypical magnocellular processing in neurotypical adults with high AQ scores. This was based on the identification of short interaction time second order kernels as of magnocellular derivation (Kaplan and Shapley, 1986) through factors of high contrast gain, saturation at high contrast, and short latency (Klistorner et al., 1997; Jackson et al., 2013). In addition, McCleery et al. (2007) showed abnormal processing of luminance contrast in infants who were at a risk for autism, suggesting an abnormal magnocellular pathway at early stages of development which could have detrimental effects on neural areas that receive input from the magnocellular pathway.

We also found group differences for longer latencies, showing shorter N4 latencies under supraliminal than subliminal condition for the Low AQ group but not for the High AQ group. This component is considered to represent event integration, according to previous studies (Liddell et al., 2004). This finding indicates that in later stages of emotion processing AQ groups differ mostly on condition effect but not on emotion effect. It also brings into attention a question about possible differences in processing of subliminal or briefly-presented face stimuli based on autistic tendencies. We could suggest that it is needed a further study that would look more carefully into the threshold of briefly presented face stimuli in people along the spectrum of autistic traits before giving conclusive answer about emotion processing in subliminal and supraliminal condition.

The present study did not find any emotion modulation on later ERP component representing an event integration stage of emotion processing. Similarly, Pegna et al. (2008) did not find an effect of emotion on the N4 component, but only an effect of stimulus duration (condition), similar to the present study. In the present study, we found emotional effect on the P3a latency. Results for this component showed shorter P3a latencies for happy compared to neutral faces in the supraliminal condition over the frontal region, but shorter P3a latencies for happy compared to neutral faces across both conditions in the central condition. This result put into the question a possible role of the P3a in the orientation stage of emotion processing, particularly showing that the P3a showed shorter latencies in supraliminal compared to subliminal condition.

Although we adopted objective criteria for visual awareness, we did not find any group differences in behavioral analysis. In addition, personal reports of participants at the end of each block revealed that they were not able to see whole faces or recognize facial expressions when faces were presented in the subliminal condition, although some of them were able to notice eyes or mouths in the subliminal condition in some of the trials. However, behavioral results showed that both subliminal and supraliminal conditions in both groups had above chance accuracy rates, with significantly larger accuracy rates for the supraliminal condition. This brings to attention research on general anesthesia where, for example, there is an important unresolved question about the possibility of partial conscious perception (Ghoneim, 2000; Daunderer and Schwender, 2004; see review in Ghoneim et al., 2009). In addition, Pessoa et al. (2005) showed in a behavioral study that when backward masking fearful facial expressions there was no universal objective awareness threshold perception of fearful emotional expressions among subjects.

In the present study, the face-specific N170 component did not show emotion discrimination either in supraliminal or subliminal condition and no group differences were observed for this component. This is in line with previous findings of absent emotional modulation for this component in a backward-masking paradigm (Kiss and Eimer, 2008). However, several backward-masking studies (Pegna et al., 2008; Smith, 2011) found that the N170 is affected by facial expressions in both subliminal and supraliminal conditions. Pegna et al. (2008) suggested that conflicting results of N170 modulation by emotional faces might be due to different references that were used. For instance, Pegna et al. (2008) and Smith (2011) used average reference whereas Kiss and Eimer (2008) used linked earlobes as a reference. In the present study we used average reference, which is suggested to be more appropriate for investigating the N170 as linked earlobes are close to the N170 location. It is important to remember that the present study examined only subjects with high and low AQ, excluding the medium AQ group, which normally makes a great part of typical population.

The results of the present study also revealed a main effect of hemisphere (i.e., right hemisphere dominance) and faster N170 latency for stimuli presented under supraliminal vs. subliminal conditions. The N170 component is usually more prominent over the right hemisphere in typically developing individuals (Rossion et al., 2003; Jacques et al., 2007; Dalrymple et al., 2011). It is interesting that the present study shows enhanced right hemisphere activation for both supraliminal and subliminal condition, indicating that the right hemisphere maintains its prominence under very briefly presented facial stimuli. We did not find any interaction of emotion and hemisphere, but Pegna et al. (2008) found enhanced N170 for fearful compared to non-fearful faces in the right hemisphere in both subliminal and supraliminal conditions.

It is easy to notice big discrepancies in results among backward masking studies. We could mention several methodological factors that can affect modulation of ERP component in these studies such as, for example: the choice of a mask, such as using a pattern by scrambling neutral faces (our study; Kiss and Eimer, 2008; Smith, 2011) or a neutral face (e.g., Liddell et al., 2004; Eimer et al., 2008; Pegna et al., 2008); an active emotion recognition task (the present study, similar to Eimer et al., 2008; Kiss and Eimer, 2008; Pegna et al., 2008; Smith, 2011) or a passive viewing task (Liddell et al., 2004), etc. We believe that our study adds some important information and understanding into subliminal face processing, including understanding of emotional processing in subclinical autistic traits. However, it is also necessary to take into consideration that the study has some methodological limitations, particularly the number of participants. Although all our participants confirmed absence of formal autism diagnosis, we had two participants with AQ score above 32, which was previously found in adults identified with autism. For example, Baron-Cohen et al. (2001) found 80% of adults with autism scored above AQ score 32, whereas only 2% of controls did so whereas Woodbury-Smith et al. (2005) found that AQ score of 32 predictably identify 76% of people diagnosed with autism when using AQ in clinical sample. However, previous research also found that scientists and those in occupations/skills such as maths, physics and engineering had higher AQ scores than the mean (Baron-Cohen et al., 1998, 2001), and many of participants of our study were students in some science and computer oriented degree.

In addition, in the light of recent findings on the P3 component, the design of the present study could be improved to include computing reaction times, as an important factor in order to measure P3 as an index of decision process due to recent findings that showed the peak latency P3 coincides with response execution (Twomey et al., 2015).

In conclusion, the present study demonstrated that individuals with low and high AQ differ in the processing of subliminal happy faces, finding increased N2 amplitude for subliminally presented happy facial expressions only in the Low AQ group (although this finding is of moderate significance, we still consider it important), but not in the High AQ group. This suggests that these differences may be based on the reduced sensitivity to social salient stimuli in individuals with High AQ.

## Author Contributions

Each author contributed to the development of the final manuscript. DPC and JC supervised this research which was part of a research PhD dissertation (SV) funded by a NHMRC grant through DPC (NHMRC project ID 1004740). DPC designed the concept proposal and JC supervised and assisted with the EEG design and analysis components. The first draft was written by SV as part of her research dissertation.

## Funding

This project was funded by the National Health and Medical Research Committee (NHMRC project ID 1004740) and the Swinburne University Postgraduate Support Scheme and the Brain and Psychological Science Research Centre support program.

## Conflict of Interest Statement

The authors declare that the research was conducted in the absence of any commercial or financial relationships that could be construed as a potential conflict of interest.
